# Referral and Management of Pretibial Lacerations in Two District General Hospitals

**DOI:** 10.7759/cureus.34231

**Published:** 2023-01-26

**Authors:** Benjamin J Jefferies, Lopa Patel, Atul Khanna

**Affiliations:** 1 Plastic Surgery, Sandwell and West Birmingham Hospitals NHS Trust, Birmingham, GBR

**Keywords:** emergency medicine and trauma, pretibial laceration, geriatric medicine, plastic and reconstructive surgery, laceration, wound repair

## Abstract

Background

Pretibial lacerations are common injuries that have a significant yet underestimated association with morbidity and mortality. Although they may occur in any age group, they are commonly followed by an often relatively minor trauma in elderly and frail patients. The six-month mortality among such patients may be more than double the age group average. Currently, 5.2 in 1000 patients per year present to the emergency departments in UK hospitals due to pretibial lacerations. The associated acute admissions have a high financial cost. Despite the significant disease burden, there is a paucity of evidence on the optimal management of such injuries. This study aimed to describe the typical demographic and injury factors of individuals presenting to two district general hospitals, as well as their subsequent management and referral.

Methodology

Relevant patients were identified through NHS coding searches. Subsequently, it was found that 99 patients presented to an NHS trust with pretibial lacerations throughout 2020. A retrospective manual evaluation of clinical documentation was performed to identify the details of the patients’ injury, management, referral, and demographics.

Results

The patients had a mean age of 55.4 (SD 28.3), and 56.6% were female. The most commonly presenting mechanism of injury was direct blunt trauma. The majority of cases were solely managed and discharged directly by the emergency department (74.8%). Of the 99 patients, 25 (25.3%) were referred to specialist services, 12 (12.1%) were managed conservatively, and 13 (13.1%) underwent operative intervention. The mean length of stay for those referred was 5.9 days, and the mean for the same was greater for those managed conservatively compared to those managed operatively (9.0 vs. 2.6 days). Among patients discharged by the emergency department, the most common method of wound closure was steristrips (n = 40; 54.1%), followed by conservative management with dressings (n = 22; 29.7%), sutures (n = 10, 13.5%) and glue (n = 5; 6.8%).

Conclusions

Overall, this study showed that the majority of patients presenting with pretibial lacerations have minor wounds that can be effectively managed in the emergency department. However, those with considerably more injuries should be provided an early referral to specialist services, where they would ideally receive early surgery and comprehensive follow-up.

## Introduction

Pretibial lacerations are common injuries with a significant yet underestimated association with morbidity and mortality. They may occur in any age group, although they present more commonly after relatively minor trauma in elderly and frail patients [1]. Elderly patients often have frail skin due to decreased dermal collagen integrity [2]. They often have numerous co-morbidities, including diabetes mellitus, peripheral vascular disease, nutritional deficiencies, and corticosteroid use, that may compromise skin integrity and delay subsequent healing [3,4]. Hematoma formation, often potentiated by anticoagulants, may compromise tissue viability through pressure effect and endothelial disruption via complex metabolic processes resulting in localized small vessel thrombosis and ischemia [5]. The six-month mortality for these patients may be more than double the age group average [6]. The current incidence rate of pretibial laceration cases in UK emergency departments (EDs) is 5.2 per 1000 patients per year, and associated acute admissions result in high financial costs [1,3,7].

Pretibial lacerations are commonly classified using the Dunkin classification [6]. Typically, class I and II injuries can be managed with simple wound closure by a non-specialist, whereas patients with class III and IV injuries should be referred for operative intervention, which is usually performed by a plastic surgery specialist [8]. However, given the demographics of the patient group, it is inevitable that some with considerable injuries will be unfit for surgical intervention. Early surgical management in suitable patients reduces wound-healing time [9]. Patients with pretibial lacerations are often neglected and postponed on surgical theatre lists as they are classified as lower priority or “the walking wounded.” These patients can experience prolonged and multiple starvation periods, which, in turn, contributes to malnutrition and often multiple comorbidities and frailty [10]. This leads to a risk of hospital-admission-related morbidity for an already vulnerable group [11].

Despite the significant disease burden, there is a relative paucity of evidence, and as such, no formal guidelines, regarding the optimal management of these patients [12]. This may result in the provision of sub-optimal management to an already morbid patient group. Further study is required to provide evidence to support the development of such guidelines. While a small number of studies have evaluated the management of inpatients for such cases, limited evidence is available for patients admitted and patients suitable for discharge from the ED.

Our study aimed to described the typical demographic and injury factors of individuals presenting to two district general hospitals and their subsequent management and referral. It also compared these aspects for patients managed in the ED and those requiring referral to specialist services, thereby contributing to the literature on these poorly studied patient groups.

## Materials and methods

Sandwell General Hospital and Birmingham City Hospital are two acute hospitals under the Sandwell and West Birmingham NHS Trust. Medical coding was performed to identify patients presenting to both hospitals with “open wound of lower leg” during 2020, from January 1, 2020, to December 31, 2020 (inclusive), which resulted in the identification of 233 patient cases. Of these cases, 30 duplicates were removed. Subsequently, after a manual evaluation of clinical documentation, 104 patients not presenting with lacerations to the pretibial area were excluded. Finally, 99 patients were included in the study.

A retrospective manual evaluation of clinical documentation was then performed to identify details of the injury, management, referral, and patient demographics. Data analysis was performed using Microsoft Excel.

Patients referred to specialist services were typically clerked by the on-call orthopedic services before subsequent management by either the plastic surgery or trauma and orthopedic teams, as appropriate.

Injuries were classified according to the Dunkin classification (Table 1).

**Table 1:** The Dunkin classification of pretibial laceration injuries [8].

Classification	Description
I	Simple laceration
II	Laceration of flap with minimal hematoma or skin edge necrosis
III	Laceration or flap with moderate to severe hematoma or necrosis
IV	Major degloving injury

## Results

A total of 99 patients were included in this study. The sample had a mean age of 55.4 years (SD 28.3), and 56.6% of them were female. The majority (80.8%) were Caucasian. Patient demographics are presented in a greater detail in Table 2, Table 3, and Table 4.

**Table 2:** Demographic details of the included patients. ED: emergency department

	Total	Discharged by ED	Referred to specialist service	Conservative management by specialist service	Operative management
Number of patients	99	74	25	12	13
Mean age	55.4	53.4	61.4	77.4	46.6
Male (%)	43.4	45.9	36.0	16.7	53.8
Female (%)	56.6	54.1	64.0	83.3	46.2
White (%)	80.8	86.5	64.0	75.0	53.8
Asian (%)	11.1	6.8	24.0	8.3	38.5
Black (%)	7.1	5.4	12.0	16.7	7.7
Mixed (%)	1.0	1.4	0.0	0.0	0.0

**Table 3:** Comorbidities of the included patients.

Comorbidity	Total	Discharged by ED	Referred to specialist services	Managed conservatively by specialist services	Operative management
Asthma	10.1	9.5	12.0	8.3	15.4
Chronic obstructive pulmonary disease	1.0	0.0	4.0	8.3	0.0
Bronchiectasis	1.0	0.0	4.0	8.3	0.0
Diabetes	5.1	5.4	4.0	8.3	0.0
Chronic kidney disease	3.0	2.7	4.0	8.3	0.0
Hypertension	23.2	21.6	28.0	50.0	7.7
Atrial fibrillation	11.1	10.8	12.0	16.7	7.7
Ischemic heart disease	6.1	5.4	8.0	8.3	7.7
Stroke	7.1	6.8	8.0	8.3	7.7
Hypothyroid	4.0	4.1	4.0	8.3	0.0
Hyperthyroid	2.0	0.0	8.0	16.7	0.0
Peripheral arterial disease	2.0	2.7	0.0	0.0	0.0
Osteoarthritis	6.1	4.1	12.0	25.0	0.0
Lymphedema	2.0	1.4	4.0	8.3	0.0
Addison’s disease	2.0	1.4	4.0	8.3	0.0
Dementia	8.1	4.1	20.0	41.7	0.0
Gout	0.0	0.0	0.0	0.0	0.0
Smoker	5.1	2.7	12.0	16.7	7.7
Cancer	3.0	2.7	4.0	8.3	0.0
Pulmonary embolism / deep vein thrombosis	2.0	0.0	8.0	16.7	0.0
HIV	1.0	1.4	0.0	0.0	0.0

**Table 4:** Regular medications of the included patients.

Medication	Total (%)	Discharged from ED (%)	Referred to specialist services (%)	Managed conservatively by specialist services	Operative Management (%)
Corticosteroids excluding inhaled ones	7.1	4.1	16.0	25.0	7.7
Inhaled steroids	10.1	9.5	12.0	16.7	7.7
Other immunosuppressants	1.0	1.4	0.0	0.0	0.0
Aspirin	8.1	5.4	16.0	25.0	7.7
Clopidogrel	6.1	6.8	4.0	8.3	0.0
Ticagrelor	0.0	0.0	0.0	0.0	0.0
Dipyramidal	0.0	0.0	0.0	0.0	0.0
Warfarin	1.0	1.4	0.0	0.0	0.0
Direct oral anticoagulant	9.1	6.8	16.0	16.7	15.4
Chemotherapy	1.0	0.0	4.0	8.3	0.0
Radiotherapy	1.0	0.0	4.0	8.3	0.0
Antiretroviral therapy	1.0	1.4	0.0	0.0	0.0

The most commonly presenting mechanism of injury was direct blunt trauma, for example. striking the pretibial area against an object or dropping an object on the shin. About 7% of the patients presented after a dog bite, which has not been specifically reported in other studies. Further, 12 (12.1%) had another concurrent injury (Table 5).

**Table 5:** Mechanism of injury of the included patients.

Mechanism	Total (%)	Discharged from the ED (%)	Referred to specialist services (%)	Conservative management by specialist services (%)	Operative management by specialist services (%)
Fall	28.3	23.0	44.0	75.0	15.4
Direct blunt trauma	46.5	52.7	28.0	16.7	38.5
Penetrating trauma	17.2	18.9	12.0	8.3	15.4
Dog bite	7.1	4.1	16.0	0.0	30.8
Road traffic collision	0.0	0.0	0.0	0.0	0.0

In most cases, injury severity was minor, with 56.6% of the cases grouped in Dunkin class I and 29.3% in Dunkin class II (Table 6). The majority of cases (74.8%) were, therefore, solely managed and directly discharged by the ED. Of the total, 25 patients (25.3%) were referred to specialist services, 12 (12.1%) were managed conservatively, and 13 (13.1%) underwent operative intervention. The mean length of stay for those referred was 5.9 days and was greater for those managed conservatively as compared to those managed operatively (9.0 vs. 2.6 days).

**Table 6:** The Dunkin classification of the included patients.

Dunkin classification	Total (%)	Discharged from the ED (%)	Referred to specialist services (%)	Managed conservatively by specialist services (%)	Operative management (%)
I	56.6	66.2	28.0	50.0	7.7
II	29.3	29.7	28.0	33.3	23.1
III	11.1	4.1	32.0	16.7	46.2
IV	3.0	0.0	12.0	0.0	23.1

Among patients discharged by the ED, the most common method of wound closure was steristrips (n = 40; 54.1%), followed by conservative management with dressings (n = 22; 29.7%), sutures (n = 10, 13.5%), and glue (n = 5; 6.8%). Concerning operative management, all 13 patients who received surgery underwent debridement, washout, and closure. Two returned to the theatre (seven and eight days later) for skin grafting. One patient underwent a surgical repair of the extensor hallucis longus and extensor digitorum tendons during the initial operation. No post-operative complications, such as graft failure, were observed in either of the skin grafting cases. Two of the operative patients received physiotherapy, both on day one post-surgery. An X-Ray was performed on 42 (42.4%) patients, with one of the cases revealing an associated fracture. A total of 30 (30.3%) patients received antibiotics, and 10 patients were documented as having wound infection. There were no documented cases of neurovascular compromise or compartment syndrome.

## Discussion

This study described the demographic details and management of patients presenting with pretibial lacerations at the ED of two hospitals. While a few studies have provided evidence on inpatient management, no study has evaluated all patients presenting with pretibial lacerations, including those managed solely by the emergency department, yet. Thus, this study makes an important contribution to the literature by describing the overall clinical burden due to this condition. The investigation of these patients is important as a majority of the patients in this study had comparatively minor injuries.

Those patients managed in the ED primarily underwent wound cleaning and primary closure. The most common method was the use of steristrips. There is no clear evidence on the best method for primary closure for these wounds, but thin and friable skin over the pretibial area may make suturing challenging. As such, methods such as steristrips or skin glue may be a more appropriate management option, especially for less severe injuries.

The demographics of patients admitted as inpatients are broadly similar to those described in previous studies [6,12]. Patients’ medications had a notable impact on the likelihood of requiring admission. Corticosteroids (16.0% vs. 4.1%), aspirin (16.0% vs. 5.4%), and direct oral anticoagulants (16.0% vs. 6.8%) were associated with an increased risk of patients requiring specialist services compared to being managed solely by the ED.

Of those patients referred to specialist services, those managed conservatively had a higher mean age (77.4; SD 21.1) vs. those managed operatively (46.6; SD 26.0). It is, therefore, possible that frailty and co-morbidities may influence the decision to manage conservatively, although it is difficult to draw meaningful conclusions from our data regarding the importance of co-morbidities and medications in such decisions. This is because of the relatively small number of patients managed operatively. Other factors such as patient preference and the exact nature of the injury also play a role in this regard. However, shorter hospital stays in operatively managed patients, along with the low incidence of postoperative complications in this study, may suggest that this is the preferable approach in those fit for surgery, which has also been indicated in the existing literature [6,9,10].

In the sample, 42.4% received an X-ray. While an element of clinical judgment is involved in choosing to image patients, given the often frail patient population and common mechanisms of injuries, we advocate the use of a low threshold for imaging to avoid concurrent bony fractures.

Similarly, the need for antibiotic therapy should also be considered. Although antibiotics are not needed in all cases, given the propensity for poor healing in patients with these injuries, it would be prudent to avoid complicating recovery with the incidence of infection due to concerns over wound contamination.

In this study, only two of the post-operative patients underwent physiotherapy. We assume that perceived “minor” injuries such as pretibial lacerations may be overlooked in the allocation of post-operative resources compared to other conditions. However, since early physiotherapy has had favorable outcomes in pretibial lacerations, this is clearly an area for improvement when considering the holistic management of these injuries [6].

This study is limited by its retrospective nature and the usage of medical coding to identify cases, as this may have resulted in some datasets being missed. It is also difficult to draw conclusions regarding long-term outcomes as patients were not formally followed up, since this study was a retrospective one, but most were routinely followed up in the community.

## Conclusions

Overall, this study showed that the majority of patients presenting with pretibial lacerations have minor wounds, which can be effectively managed in the ED. However, in those with more considerable injuries, we advocate an early referral to specialist services, where patients would ideally receive early surgery and comprehensive follow-up, as clinically appropriate. In the future, clear clinical guidelines need to be developed for the optimal management of these injuries, which are associated with considerable morbidity, mortality, and financial cost. Further publication of research addressing the paucity of evidence on this important topic is, therefore, essential.

## References

[1] Cahill KC, Gilleard O, Weir A, Cubison TC (2015). The epidemiology and mortality of pretibial lacerations. J Plast Reconstr Aesthet Surg.

[2] Shuster S, Black MM, McVitie E (1975). The influence of age and sex on skin thickness, skin collagen and density. Br J Dermatol.

[3] Gantwerker EA, Hom DB (2011). Skin: histology and physiology of wound healing. Facial Plast Surg Clin North Am.

[4] Molnar JA, Underdown MJ, Clark WA (2014). Nutrition and chronic wounds. Adv Wound Care (New Rochelle).

[5] Glass GE, Nanchahal J (2012). Why haematomas cause flap failure: an evidence-based paradigm. J Plast Reconstr Aesthet Surg.

[6] Glass GE, Jain A (2014). Pretibial lacerations: experience from a lower limb trauma centre and systematic review. J Plast Reconstr Aesthet Surg.

[7] Thomson WL, Pujol-Nicolas A, Tahir A, Siddiqui H (2014). A kick in the shins: the financial impact of uncontrolled warfarin use in pre-tibial haematomas. Injury.

[8] Dunkin CS, Elfleet D, Ling C, Brown TP (2003). A step-by-step guide to classifying and managing pretibial injuries. J Wound Care.

[9] Tuboku-Metzger V, Chambers J, Osmani O, Nightingale P, Eltigani T, Skillman JM (2014). Early debridement reduces time to healing in elderly patients with pretibial injury. J Plast Reconstr Aesthet Surg.

[10] Lo S, Hallam MJ, Smith S, Cubison T (2012). The tertiary management of pretibial lacerations. J Plast Reconstr Aesthet Surg.

[11] Creditor MC (1993). Hazards of hospitalization of the elderly. Ann Intern Med.

[12] Singh P, Khatib M, Elfaki A, Hachach-Haram N, Singh E, Wallace D (2017). The management of pretibial lacerations. Ann R Coll Surg Engl.

